# Association between fetal growth restriction and polymorphisms at sites -1 and +3 of pituitary growth hormone: a case-control study

**DOI:** 10.1186/1471-2393-5-2

**Published:** 2005-02-03

**Authors:** Ronald M Adkins, Caroline Campese, Rehana Vaidya, Theonia K Boyd

**Affiliations:** 1 Children's Foundation Research Center and Center of Genomics and Bioinformatics, University of Tennessee Health Science Center, Memphis, TN, USA; 2 Department of Biology, University of Massachusetts, Amherst, MA, USA; 3 Department of Pathology, Children's Hospital Boston, Boston, MA, USA

## Abstract

**Background:**

Fetal growth restriction is associated with significantly increased risks of neonatal death and morbidity and with susceptibility to hypertension, cardiovascular disease and NIDDM later in life. Human birth weight has a substantial genetic component, with at least a quarter of the variation attributable to additive genetic effects.

**Methods:**

One hundred twenty-five subjects (83 control and 42 case) were selected using stringent inclusion/exclusion criteria. DNA sequencing was used to identify 26 single nucleotide polymorphisms in the pituitary growth hormone gene (GH1) at which all subjects were genotyped. Association with fetal growth restriction was tested by logistic regression for all sites with minor allele frequencies greater than 5%.

**Results:**

Logistic regression identified significant association with fetal growth restriction of C alleles at sites -1 and +3 (relative to the start of transcription) that are in complete linkage disequilibrium. These alleles are present at higher frequency (6% vs. 0.4%) in fetal growth restricted subjects and are associated with an average reduction in birth weight of 152 g in normal birth weight and 97 g in low birth weight subjects.

**Conclusions:**

There is suggestive association between fetal growth restriction and the presence of C alleles at sites -1 and +3 of the pituitary growth hormone gene.

## Background

Fetal growth restriction (FGR) is a major risk factor for illness in the perinatal period and throughout life, with the smallest 7.5 percent of infants accounting for two-thirds of infant deaths [1]. Term, low birth weight infants are at least five times more likely to die in the first year [2,3] and are second only to premature infants in their rates of morbidity and mortality [4]. FGR infants have an increased frequency of hypoglycemia, hypothermia, polycythemia, neurodevelopmental deficits, and cerebral palsy [5]. Later in life, individuals born FGR are at elevated risk of hypertension, cardiovascular disease, and non-insulin dependent diabetes [6]. For example, FGR increases the risk for adult onset of non-insulin dependent diabetes two- to three-fold [7,8]. The ability to both diagnose and treat FGR early in gestation has enormous potential to reduce childhood and adult illness.

It is difficult to distinguish the genetic and environmental components of human birth weight variation, but recent studies support a major genetic component to birth weight variation. Clausson et al.'s [9] study of the offspring of female dizygotic and monozygotic twins (2,009 twin pairs) estimated heritability for human birth weight of 42%, although the confounding influence of shared environmental effects must be considered. In a study of 3,562 captive macaques that minimized environmental heterogeneity, Ha et al. [10] estimated a total heritability for birth weight of 51%, with an additive genetic component of 23%. These findings demonstrate that comparatively simple and readily identifiable genetic factors influence birth weight. In concert with this recent research, FGR tends both to cluster in families and to recur in successive generations [11-14].

The five genes of the human growth hormone locus reside within about 45 kb on chromosome 17 [15]. Pituitary growth hormone (GH1) is by far the most thoroughly studied of the genes and lies at the 5' end of the cluster. The remaining four genes, placental growth hormone (GH2) and three chorionic somatomammotropins (CS1, CS2, and pseudogene CS5 or CSHP1), are expressed only from the placenta. The promoter region of GH1 is unusually polymorphic, with 16 SNPs having been identified in a span of 535 bp [16-18]. Most of these SNPs occur at the comparatively small number of sites that exhibit sequence differences among the five genes of the GH locus, and this has been interpreted as evidence of gene conversion [16,19].

Here we use DNA sequencing to identify and to determine the frequencies of both 12 newly-identified single nucleotide polymorphisms (SNPs) in the promoter and coding region of the GH1 gene and 15 previously reported SNPs [16,18]. Using a case-control design, we identify two SNPs in complete linkage disequilibrium near the start of transcription of the GH1 gene that may predispose to reduced birth weight.

## Methods

### Human subjects

DNA was extracted from placental tissue from 125 live births (83 normal birth weight, 42 low birth weight) at Baystate Medical Center (Springfield, MA) in a case-control study of the genetic predisposition to fetal growth restriction. All subjects were Caucasian, both Hispanic and non-Hispanic. Classification of newborns as fetal growth restricted (or IUGR, intrauterine growth retarded) followed the definition of the American College of Obstetricians and Gynecologists as those newborns below the 10^th ^percentile of size for gestational age [20]. Our cut-off of 2,500 g at term (>37 weeks gestation) corresponds to the lowest 7.5 percentile of all US Caucasian deliveries, or the lowest 10^th ^percentile of female and lowest 6.5 percentile of male deliveries [21]. Stringent inclusion/exclusion criteria (Table 1) were employed by a placental pathologist (TKB) to reduce nongenetic contributors to birth weight variation. Ethical approval to conduct this study was obtained from the Human Subjects Institutional Review Board of the University of Massachusetts.

**Table 1:** Inclusion and exclusion criteria for the study of fetal growth restriction

Inclusion Criteria	
	≥ 37 weeks gestation (full-term)
	Mother 17–35 years old
	Singleton pregnancy
	Case (fetal growth restricted): <2,500 g birth weight
	Control: 3,000 to 4,000 g birth weight
Exclusion Criteria	
	Karyotypic abnormalities, including confined placental mosaicism
	Placental abnormalities
	Birth defects or syndromic conditions (i.e., Silver-Russell Syndrome)
	Pregnancy complications
	Preeclampsia
	Type 1, Type 2 or gestational diabetes
	Meconium staining
	Uterine infection
	Maternal chronic illnesses (i. e., hypertension, AIDS, hepatitis, endocrinological)
	Known illicit drug use
	Rh disease

### Polymerase chain reaction (PCR) and sequencing

The region from -624 (relative to the start of transcription; GenBank accession J03071) to +1,726 (197 nucleotides after the termination codon) of GH1 was amplified in two overlapping fragments: -624 to +541 (GHN-1F 5' AGGGACCTGGGGGAGCCCCAGCAA 3', GHN-1R 5' TCACCCCTTCCTGCCACCCCTGAT 3') and +450 to +1,726 (GHN-2F 5' CCATCGTCTGCACCAGCTGGCCTT 3'; GHN-2R 5' GCCCTACAGGTTGTCTTCCCAACT 3'). Approximately 50 ng of DNA was used as a template in a polymerase chain reaction with 30 cycles of 95°C (1 minute), 62°C (1 minute), and 72°C (2 minutes 30 seconds). PCR products were purified from agarose using a QIAquick PCR Purification kit (Qiagen) and sequenced directly with BigDye v2.0 chemistry (Applied Biosystems) and run on either an ABI Prism 377 or 3100 automated DNA sequencer. PCR products were sequenced with the PCR primers and additional internal primers: 5' AAGCACAGCCAATAGATTG 3', -459 to -441; 5' GCACAAGCCCGTCAGTGGCC 3', -108 to -89; 5' GGATTTTAGGGGCGCTTACC 3', +71 to +90; 5' CATCTCCCTGCTGCTCATC 3', +931 to+949; 5' GCGCTTGGGYACTGTTCCCT 3', +1280 to +1299.

### Single nucleotide polymorphism (SNP) genotyping

Sequence traces were aligned and assembled into contigs by the program Polyphred [22]. Contigs were viewed in either the program Consed [23] or Sequencher (Gene Codes Corp.) and polymorphisms confirmed visually. Twenty-six polymorphic sites were identified (Table 2), including all of the sites identified by Horan et al. [18] in 154 British military recruits with the exception of site -339 which had a minor allele (deletion of G) frequency of 3.6% in their study.

**Table 2:** Frequency of alleles at 26 single nucleotide polymorphisms in the promoter and coding region of pituitary growth hormone and the nucleotide present at the homologous site in other members of the human GH locus

		Frequency		GH1 paralogs					
					
Position*	Alleles	This Study	Horan et al.	CS-5	CS-1	GH2	CS-2	Categorization	Function**
-580	A	0.985		A	A	A	A	Constant	
	G	0.015							
-476	A	0.012	0.013	A	G	A	G	Variant	
	G	0.988	0.987						
-360	A	0.972		G	G	G	G	Variant	
	G	0.028							
-352	G	0.012		T	G	G	G	Variant	
	T	0.988							
-308	G	0.732	0.753	T	C	T	C	Variant	
	T	0.268	0.247						
-301	G	0.732	0.753	T	T	T	T	Variant	
	T	0.268	0.247						
-278	G	0.628	0.601	T	A	T	A	Variant	NF1
	T	0.372	0.399						
-168	C	0.024	0.019	T	C	T	C	Variant	
	T	0.976	0.981						
-75	A	0.900	0.886	G	A	G	A	Variant	PIT-1
	G	0.100	0.114						
-57	G	0.687	0.633	G	T	A	T	Variant	Vitamin D Receptor
	T	0.313	0.367						
-31	G	0.882	0.867	G	G	-	G	Variant	Vitamin D Receptor
	-	0.118	0.133						
-6	A	0.565	0.588	A	G	A	G	Variant	Transcription Start
	G	0.435	0.412						
-1	A	0.847	0.932	C	T	A	T	Variant	Transcription Start
	C	0.044	0.003						
	T	0.109	0.065						
3	C	0.044	0.003	C	G	G	G	Variant	Transcription Start
	G	0.956	0.997						
16	A	0.976	0.981	G	A	A	A	Variant	5' UTR
	G	0.024	0.019						
25	A	0.980	0.981	C	A	A	A	Variant	5' UTR
	C	0.020	0.019						
59	G	0.072	0.049	G	G	G	G	Variant	5' UTR
	T	0.928	0.951						
69	A	0.968		G	C	G	G	Variant	Thr/Ala
	G	0.032							
124	A	0.988		G	A	G	A	Variant	Intron
	G	0.012							
128	A	0.988		C	C	T	C	Variant	Intron
	T	0.012							
140	A	0.004		G	G	G	G	Constant	Intron
	G	0.996							
144	A	0.012		G	G	G	G	Constant	Intron
	G	0.988							
281	C	0.024		T	C	C	C	Variant	Intron
	T	0.976							
596	C	0.986		T	T	T	T	Variant	Intron
	T	0.014							
1070	A	0.004		G	G	G	G	Constant	Synonymous
	G	0.996							
1169	A	0.331		T	T	T	T	Constant	Intron
	T	0.669							

* Relative to the start of transcription

** Polymorphisms in known transcription factor binding sites are shown. Site +69 is part of the signal peptide.

### Statistical analyses

The five genes of the human growth hormone locus exhibit high sequence similarity, and the paralogous regions corresponding to the portion of GH1 sequenced in this study (-624 to +1,726) were multiply aligned. Nucleotide positions (Table 2) were designated as invariant if all five genes had the same nucleotide or the four paralogs of GH1 were identical and matched the major allele at that site in GH1. This categorization explicitly assumes that only the minor alleles in GH1 are the product of gene conversion and that minor alleles not observed in paralogs of GH1 are the result of unique mutations. The proportion of GH1 polymorphisms at invariant versus variant sites was compared by Fisher's Exact Test to determine if there was an over-representation of polymorphic sites among variant sites.

Logistic regression, using FGR status as the outcome, was performed on gestational age and genotypes at sites with a minor allele frequency above 5% (Table 3). Sites -301 and -308 (relative to the start of transcription) are in complete linkage disequilibrium and the minor C alleles at sites -1 and +3 are in complete linkage disequilibrium. Therefore, sites -301 and +3 were excluded from logistic regression to avoid multicollinearity. Based on the results from logistic regression, separate ANOVA (Table 4) was performed on gestational age and the AA versus AC genotypes at site -1 within low birth weight and within normal birth weight subjects. Empirical p values for the F statistic for the genotypic effect, corrected for multiple comparisons, were determined by 2,000 random permutations of the genotypic data [24]. All statistical analyses were performed using the Stata program (Stata Corp., College Station, TX).

**Table 3:** Logistic regression on FGR status based on gestational age and SNP genotype for GH1 polymorphisms with minor allele frequency greater than 5%

Variable	Odds Ratio	95% CI	Z Score	P-value
Gestational Age	0.42	0.26–0.66	-3.75	<0.001
-308	2.66	0.54–13.19	1.20	0.23
-278	1.62	0.27–9.80	0.52	0.60
-75	1.70	0.55–5.23	0.93	0.35
-57	3.34	0.62–18.12	1.40	0.16
-31	0.85	0.27–2.65	-0.29	0.78
-6	2.11	0.66–6.74	1.26	0.21
-1 A/T	0.76	0.23–2.44	-0.47	0.64
-1 A/C	0.10	0.01–0.77	-2.21	0.03
+59	1.48	0.32–6.95	0.50	0.62
+1169	1.05	0.38–2.95	0.10	0.92

**Table 4:** ANOVA on A/C genotypes at site -1 and gestational age in normal and low birth weight subjects

	Normal Birth Weight		Low Birth Weight	
		
	Site -1	Gestational Age	Site -1	Gestational Age
Mean AA	3382.8		2287.9	
Mean AC	3230.2		2190.4	
ANOVA F	3.75	4.55	3.12	2.25
P-value	0.056*	0.002	0.073*	0.099

* Empirical P value corrected for multiple testing determined by 2,000 random permutations of the genotypic data [24]

## Results

### Polymorphism in the GH1 gene

Among the 125 subjects sequenced from -624 to +1,726 of the GH1 gene, 26 polymorphic sites were identified (Table 2). These included all but one of the 15 sites characterized by Horan et al. (2003). In the region of overlap between the two studies, we failed to detect variation at site -339, where there is a minor allele deletion of a single nucleotide with frequency 3.6% in British army recruits, and identified two additional variants at sites -360 and -352 with minor allele frequencies 2.8% and 1.2%, respectively. Therefore, the discrepancies between the two studies in the identification of SNPs can most likely be ascribed to sampling error. Outside the region surveyed by Horan et al. (2003), we detected 10 additional polymorphisms. All of these had minor allele frequencies ≤ 3.2%, except an intron four polymorphism at site +1169 with a minor allele frequency of 33.1%. In general, polymorphisms in the promoter of GH1 tend to be more densely clustered and exhibit higher minor allele frequencies than in the transcribed region.

### Evidence of gene conversion

An alignment of the region of GH1 sequenced in this study with the paralogous sequences of placental growth hormone and chorionic somatomammotropins indicated that there are 1,979 invariant sites and 293 variant sites (excluding 78 sites that could not be unambiguously aligned), as defined in the methods. Of the invariant sites, 5 are polymorphic in GH1, while 21 of the variant sites are polymorphic. A comparison of the proportion of polymorphic sites at invariant and variant sites by Fisher's Exact Test is highly significant (P <<0.001). This result indicates that there is a strong bias for polymorphisms in GH1 to occur at the minority of sites that exhibit sequence divergence among the paralogous genes. The high correspondence between the sequence of minor alleles in GH1 and nucleotides present in paralogs of GH1 supports previous assertions that the unusually high polymorphism of the GH1 gene is driven by gene conversion [16,18].

A proportion of this bias may be explained by selective constraint, in that sites that are polymorphic within GH1 may be under less selective constraint and thus more free to exhibit sequence divergence among paralogs. However, to entirely explain the bias towards polymorphism at sites of divergence, one must assume that about 1,804 (91%) of the invariant sites are selectively constrained and not free to vary. Given that a substantial proportion (814 bp, ~36%) of the sequence surveyed in this study is composed of introns, this assumption seems unreasonable.

### Association with fetal growth restriction

Logistic regression (Table 3) was performed on gestational age and SNPs with minor allele frequencies greater than 5%, excluding sites -301 and +3 because they are in complete linkage disequilibrium with other sites, to identify associations with fetal growth restriction. Gestational age was significant because the FGR subjects exhibit a slightly younger average estimate of gestational age (38.1 vs. 39.1 weeks, t = 4.9, p < 0.0001; gestational ages rounded to nearest week). However, even accounting for the effect of gestational age, the C allele at site -1 was significantly associated with FGR in the combined regression. However, this allele did not retain significance in a regression on only gestational age (p < 0.001) and the A/C polymorphism at site -1 (p = 0.242).

Although the A/C polymorphism at site -1 was not significant in a reduced model, we decided to investigate this site for three reasons. First, the C allele at site -1 is in complete linkage disequilibrium with C at site +3. Second, both of these sites are located at the start of transcription, making them good candidates for affecting the level of transcription of GH1. Third, the only paralog that shares C at these sites is CS-5, a pseudogene, consistent with the possibility that C at sites -1 and +3 is disadvantageous. The C allele at both sites exhibits a much higher frequency in low birth weight (6%) versus normal birth weight (0.4%) subjects. Restricting examination only within the normal or low birth weight subjects, the AC genotype is associated with an average reduction of birth weight of 152 g and 97 g in normal and low birth weight subjects, respectively. However, this difference does not achieve significant in an ANOVA on the A/C polymorphism and gestational age (Table 4).

## Discussion

Birth weight in humans and other primates exhibits substantial heritability [9-13]. Although a suite of environmental and genetic/karyological insults are known to cause fetal growth restriction, perhaps as many as 40% of FGR cases have no known etiology [25]. Therefore, identification of the underlying genetic variants that predispose to FGR could have significant medical significance if it allows us to identify early in gestation those pregnancies that are at increased risk of growth retardation. A logical place to begin such a search is among those genes that are known to be major regulators of fetal growth and to exhibit significant differences in circulating protein concentrations between normal and FGR pregnancies, such as the members of the growth hormone-prolactin and insulin-IGF families of hormones, receptors, and binding proteins. Here, we use a stringently selected set of subjects to report suggestive association of SNPs in GH1 with fetal growth restriction.

Adjusted for gestational age, the C alleles at sites -1 and +3 of the GH1 gene appear to be associated with reduction in birth weight. The marginal significance of these results may be the result of several factors. First, we examined 125 total subjects, roughly one-third of them FGR, and this number may give inadequate statistical power to identify weak genetic effects. Second, the C allele is low frequency, providing a small number of heterozygotes for the allele and no homozygotes. It is worth noting that among normal birth weight subjects we observed a very similar frequency for the C allele as Horan et al. (2003) did among British army recruits (0.4% vs. 0.3%) but that among the low birth weight subjects the frequency of the C allele is substantially higher (6%). Comparing all the other allele frequencies (Table 2) between the two studies of Caucasian populations, no other allele shows such a large magnitude of difference, although the proportional difference in frequency of C at -1 and +3 may be somewhat distorted by sampling error at low allele frequencies. Third, FGR undoubtedly has a heterogeneous genetic etiology, and it may be unlikely to find any genetic variant that accounts for more than a small proportion of cases.

If the C allele at -1 and +3 exhibits true association with FGR, it unfortunately may be very difficult to determine if the effect is due to one or both sites because they are in complete linkage disequilibrium. Nevertheless, the presence of both alleles at the start of GH1 transcription provides both variants with a biologically plausible possibility to affect the level of transcription of GH1. Future work should be devoted to examining the effect of these alleles on transcription levels both alone and in combination. Site -1 is somewhat unique among single nucleotide polymorphisms in that three alternate alleles exist at that site (the major allele A and the minor alleles C and T). There are two possible explanations for this observation. First, site -1 could be a hotspot for nucleotide mutation, with transversions from A to C/T occurring often. Second, GH1 may be the recipient of gene conversion events from more than one paralog within the GH locus. Among the four other genes of the GH locus, all three alleles observed at site -1 occur (Table 2). Given the predominant effect that gene conversion [16,18] appears to have on the patterns of nucleotide polymorphism in the GH1 gene, the latter explanation may be more plausible.

It must be pointed out that the common wisdom is that GH1 plays no role in regulating fetal growth because the GH receptor is expressed fairly late in gestation and because anencephalic infants or those born without a pituitary achieve nearly normal length [26]. Importantly, we restricted our study to full term deliveries. It is possible that late in gestation, when fetal GH1 is expressed and GHR receptors are present in a wide variety of fetal tissues, pituitary growth hormone begins to have a growth stimulatory role sufficient to account for the 90–150 g difference in birth weight between genotypes for the A/C polymorphism at -1 and +3 that we observed.

## Conclusions

In a stringently selected set of subjects, C alleles at sites -1 and +3 relative to the start of transcription of GH1 have a higher frequency (0.4% vs. 6%) in fetal growth restricted newborns. These two alleles are in complete linkage disequilibrium and their presence is associated with a reduction in birth weight of 152 g in term, normal birth weight subjects and 97 g in term, low birth weight (<2,500 g) subjects. In combination with environmental, behavioural and other genetic factors, these alleles may contribute to fetal growth restriction.

## Competing interests

The author(s) declare that they have no competing interests.

## Authors' contributions

RMA conceived the project and directed its design and execution. CC and RV performed the molecular genetic work and participated in preliminary analyses. TKB performed the subject selection.

## Pre-publication history

The pre-publication history for this paper can be accessed here:


